# Race, nationality, and partisanship shape U.S. public support for climate disaster aid: Evidence from two survey experiments

**DOI:** 10.1371/journal.pone.0347292

**Published:** 2026-05-06

**Authors:** Volha Charnysh, Jared S. Kalow, Evan S. Lieberman, Erin E. Walk

**Affiliations:** 1 Department of Political Science, Massachusetts Institute of Technology, Cambridge, Massachusetts, United States of America; 2 Annenberg School for Communication, University of Pennsylvania, Philadelphia, Pennsylvania, United States of America; Université Paris Dauphine: Universite Paris Dauphine, FRANCE

## Abstract

We examine how race, nationality, and political partisanship influence U.S. public support for climate-related disaster aid. Using two preregistered survey experiments (N = 7,511), we varied the race (Black/White) and nationality (U.S./Brazil or South Africa) of flood victims depicted in an artist’s rendering of a fictional disaster. Respondents were significantly less supportive of aid to Global South victims than U.S. victims, with the gap largest among Republicans. Race effects were smaller and context-dependent: domestically, White Republicans expressed less generosity towards Black than White victims, while White Democrats showed the opposite tendency. Our analysis provides suggestive evidence that perceptions of social distance and deservingness shape willingness to provide climate aid across racial and national lines.

## Introduction

The extent to which citizens are willing to support victims of natural disasters is a core dimension of pro-social behavior. As climate change increases the frequency and severity of damaging storms, identifying the factors that shape individual generosity toward disaster victims has become an increasingly urgent scholarly task. A growing body of research has shown that race and racial resentment shape attitudes towards *domestic* disaster aid [e.g., 1, 2, 3], but little is known about how race operates with respect to attitudes about international aid [4]. Only two recent studies address this question directly, with divergent findings. Using a survey experiment, [5] shows that White Americans are *more* supportive of foreign aid to majority-Black countries than majority-White countries because they perceive the former as less capable of agency. In an observational setting, [6] find that White Americans with higher racial resentment are *less* supportive of foreign aid.

We advance this literature by directly comparing the effects of disaster victims’ national and racial identity on support for governmental and private aid and by considering a broader range of mechanisms shaping donor generosity in two original surveys in the United States. Moreover, we consider how these patterns may vary conditional on the partisan attachment of individuals.

We hypothesize that Americans will be less generous when disaster victims are foreigners and that for White respondents, the effects of victims’ race will be moderated by nationality. Whereas research on domestic redistribution has focused on racial resentment as the key factor, we evaluate several additional mechanisms relevant to prosocial behavior: perceived social proximity, paternalistic sentiment toward the victims, and ability to understand victims’ emotional state. We hypothesize that White Americans’ willingness to assist racial outgroups in the U.S. and the Global South is driven by different considerations due to the significant geographic, institutional, and economic barriers that separate them. In particular, under conditions of low outgroup threat and stable intergroup inequality, White respondents may see Black victims in the Global South as “warm” but lacking in capacity to deal with their problems, leading them to support greater assistance, albeit of a more paternalistic kind [5,7].

We expect the effects of nationality and race on support for governmental assistance to be larger among Republicans, in line with earlier work that suggests U.S. Republicans are more likely to endorse a more exclusionary and restrictive vision of national identity than Democrats [8]. We further expect Republicans to be less generous toward victims overall because conservative ideology emphasizes individual responsibility and self-reliance [9], following patterns in earlier work [10,11].

We test these hypotheses in two separate surveys (N = 5,005 and N = 2,506) conducted in 2023 and 2024, preregistered at the Open Science Framework (OSF) at https://osf.io/a3c6f and https://osf.io/b8e5t. Study 2 was conducted to replicate the results from Study 1, with a slightly different treatment in order to boost external validity, and included additional questions.

Overall, we find that respondents were significantly less supportive of aid to Global South victims than U.S. victims, with the gap largest among Republicans. Race effects were smaller and context-dependent: Republicans expressed less generosity towards Black than White victims based in the U.S., while Democrats showed the opposite tendency. There were no statistically significant differences in generosity toward Black and White victims based in the Global South.

## Theoretical framework

Advocacy campaigns routinely urge Global North citizens to assist distant strangers, emphasizing universal moral obligations [12,13]. Yet behavioral research shows that people privilege variously defined ingroup members when distributing resources and that their generosity declines with physical and social distance [14–16]. Across various situations, ingroup attribution bias leads people to perceive ingroup members as suffering through no fault of their own and therefore more deserving of assistance than outgroup members, whose challenges are attributed to personal failings [17]. People are also more likely to accurately perceive ingroup members’ emotional states, increasing empathetic reactions [18–20].

Researchers have further shown that people do not treat all outgroups in the same way, although mechanisms responsible for these differences are less well understood. One prominent model organizes outgroup stereotypes along the dimensions of warmth and competence [21]. In this model, ingroup members are viewed as high on both dimensions, while outgroups fall into the three remaining categories, associated with distinct emotional responses. High-warmth and low-competence outgroups evoke paternalistic prejudice (pity), low-warmth and high-competence individuals evoke envious prejudice, and low-warmth and low-competence individuals evoke disgust [22]. The stereotype-content model is helpful for understanding relative valuations of outgroups and can explain why some outgroups may receive more resources than others. For instance, researchers find that high warmth stereotypes increase active helping behavior, while high competence stereotypes encourage passive cooperation [23,24]. Most relevant to this study, high-warmth and low-competence outgroups “are often helped instead of disregarded” and are therefore treated better than low-warmth and low-competence outgroups [24]. However, the help they receive is more likely to be dependence-oriented: for example, it may involve in-kind, prepackaged goods rather than unconditional cash transfers and therefore does not account for recipients’ preferences [25–27].

The choice of outgroup stereotype is linked to levels of intergroup competition and differences in social status: groups that compete with the ingroup for resources are stereotyped as low in warmth, while high-status groups are seen as more competent and capable [28,29]. In the domestic context, welfare recipients are often stereotyped as low in both warmth and competence [29,30].

In our study, two central categories that structure prosocial behavior are nationality and race. Repeated cross-national surveys show Americans prioritize domestic spending over foreign aid and condition support for international assistance on domestic trade-offs, consistent with a nationality/belonging bias in perceived deservingness [31]. In line with this research, we expect that Americans will be more willing to assist a U.S. family suffering from climate-induced flooding than a similar family in the Global South.

Race has been studied primarily in the context of domestic redistribution. A large body of work in U.S. politics shows that U.S. Whites perceive Black Americans as less deserving of assistance [1,32,33]. Consistent with group attribution bias, the media portrays African Americans as responsible for their own problems and unjustly benefiting from government support [32,34]. Most notably, [1] demonstrate that racial resentment, which taps sentiments about Black Americans’ motivation and work ethic, is a powerful predictor of White Americans’ preferences for disaster spending. We thus expect White Americans to be less generous toward Black Americans than toward White Americans.

At the same time, research shows that the effects of racial bias depend on contextual cues and individual predispositions. For example, Huddy and Feldman [35] show that racial cues influence Whites’ support for scholarships only when paired with class signals and are moderated by racial resentment. Research on charitable giving arrives at similar conclusions. Fong and Luttmer [36] find that feeling close to one’s racial group boosts giving, but racial cues alone (e.g., photos of Black victims of Hurricane Katrina) do not. Likewise, Fong and Luttmer [37] and Gross and Wronski [38] report null average effects of race on donations, but show that race systematically shapes perceptions of victims’ deservingness.

Evidence of bias in favor of co-racial foreigners has been largely restricted to contexts where foreigners are physically present and compete for local resources. For instance, Global North citizens express greater support for refugees who share their race or religion and discriminate against non-Whites and Muslims because they perceive the latter as a cultural threat [39–41]. One exception is [5], who shows that paternalistic prejudice leads American Whites to support greater assistance to majority-Black than majority White countries. Consistent with the stereotype-content model, Western media frequently portrays residents of Black-majority countries as likable but helpless victims of violence, poverty, and natural disasters, i.e., as warm but incompetent [5]. Because racial outgroups in the Global South pose minimal threat due to geographic distance, institutional barriers, and enduring global inequalities, they may elicit paternalistic prejudice rather than racial resentment, leading to pity-based, dependency-oriented help.

We thus consider two competing hypotheses about the effect of victims’ race in the Global South on support for assistance. First, White Americans may be less generous toward foreign Black than foreign White victims because they view them as less deserving, mirroring domestic racial bias. Alternatively, Whites may be more generous toward foreign Black victims because they perceive them as non-threatening (i.e., warm) but also low in competence and thus in need of (paternalistic) assistance. This alternative suggests that racial prejudice may take a different form in international contexts, emphasizing paternalism rather than resentment.

### Heterogeneous effects by party

We expect political ideology to moderate the effects of nationality and race cues on support for aid.

Studies of American citizens found that Republicans are more likely to endorse an exclusionary and restrictive conception of nationhood than Democrats [8] and have higher levels of anti-Black prejudice [35,42,43]. If this is the case, race and nationality primes should have larger effects on Republican respondents’ generosity.

American citizens tend to differ in their view of the role of government in domestic and international redistribution according to the political party that they support. Republicans are more likely to emphasize self-reliance and individual responsibility than Democrats, which has been used to explain their lower support for domestic welfare programs [35,44,45]. Consistent with ideological differences, [3] show that Republicans prefer market-based disaster aid to direct federal aid. Republicans are also less supportive of foreign aid than Democrats [10,11] and more likely to prioritize aid aligned with strategic interests rather than aid designed to reduce inequality [46]. This leads us to expect lower levels of support for climate assistance among Republicans than Democrats across experimental conditions.

## Methods

To understand how Americans respond to the needs of national and racial outgroups affected by climate change we conducted two surveys, on August 18–24, 2023 (N = 5,005) and on February 12–16, 2024 (N = 2,506). For both surveys, we recruited participants aged 18 and above, using Prolific, a research-focused online survey platform whose respondents outperform those on other platforms and student samples in attention, data quality, and compliance [47]. Potential respondents were told they would participate in a 10-minute online survey about their views about a number of challenges facing the U.S. government.

Our study was determined to be exempt from full review of the Massachusetts Institute of Technology Institutional Review Board (Committee on the Use of Humans as Experimental Subjects, exempt study 4863), as it involved a benign intervention and did not put subjects at risk. Voluntary informed consent was obtained from all participants. Participation was not compensated.

As shown in the supporting information S1 File, our samples are broadly representative of the U.S. population in gender and region, but are more educated and Democratic than the national average. These characteristics of the sample bias against finding significant effects of nationality and race treatments in the full sample because more educated and liberal Americans hold lower levels of outgroup prejudice [43,48].

We first asked respondents about their racial identity. A randomly selected half of the respondents was presented with a feeling thermometer toward foreigners and various racial and socioeconomic groups. The other half received these questions at the end of the survey to avoid priming effects.

**Fig 1:**
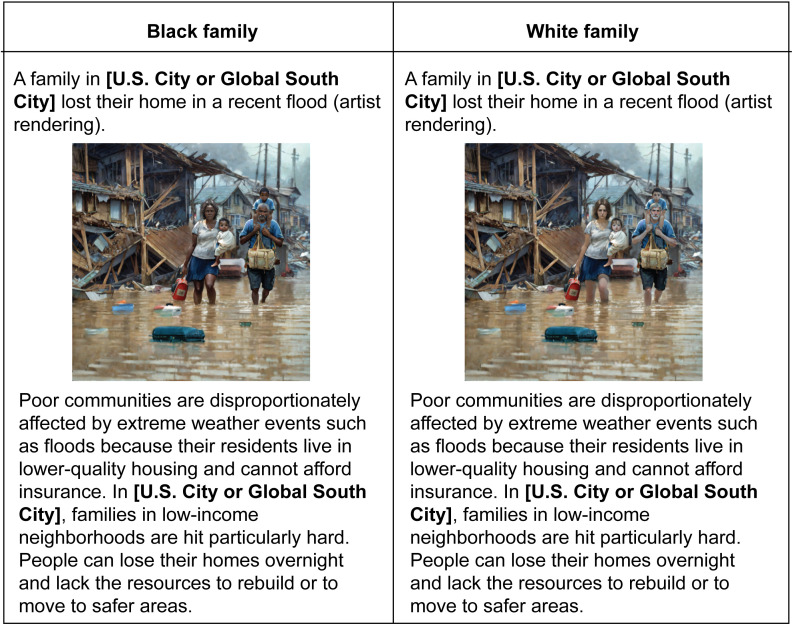
Description of four treatment arms. Each respondent was shown one of two images — depicting either a White family or a Black family — and told that a flood had occurred in either the United States or a Global South country (Brazil in Study 1; South Africa in Study 2). Illustrations courtesy of Talha Sattar.

Next, respondents were presented with a vignette about a family who lost their home in a recent flood and told that poor communities are disproportionately affected by extreme weather events and that their residents live in lower-quality housing and cannot afford insurance (see Fig 1).

Our experimental design involved varying a few details of the vignette. Specifically, we used a 2x2 factorial design in which respondents were experimentally assigned to (1) an image of either a White or Black family. We deviated from [5], who primed race using country names, to isolate the effects of race from country characteristics, such as level of development, corruption, relationship with the U.S., and location in the Global South, and (2) being told that the family lives in the U.S. or a country in the Global South (Brazil in Study 1 and South Africa in Study 2).

We selected these two countries because they are developing democracies, frequently affected by climate-related disasters, and share with the United States high levels of racial diversity and socio-economic hierarchies [49] in which race and class largely overlap. In turn, it was plausible to show audiences victim families with similar features that appear as White or Black and coming from any of the three countries. This allows us to consider whether American audiences may perceive racial identity, social proximity, and victim competence differently depending on the national context that is experimentally assigned. While this is a strength for the internal validity of the research design, it is potentially a limitation on external validity, as many countries that might receive development assistance from the United States have distinct demographics and reputations among the American public. Of course, any choice would pose some limits in this regard, but the fact that we recovered similar results with two different country cases is somewhat reassuring for the robustness of the findings.

We thus consider a total of four main experimental conditions: White U.S., Black U.S., White Global South (G.S.), and Black G.S. flood victim families. Within each country condition, we also randomized which city the family was from to test for potential associations with a particular city (Charleston, SC and Jacksonville, FL in the U.S. treatment, Campo Grande and Duque de Caxias in the Brazil treatment, and Port Elizabeth and Gqeberha in the South Africa treatment). City choices did not affect the results. Our research design thus allows us to directly compare generosity toward foreign and American victims, holding race and perceived need constant, and to examine whether the effects of race vary with nationality.

Two features of our design are important to highlight. First, we opted for a commissioned artist’s rendering of a hypothetical family as opposed to using photographs of real-life families to avoid engaging in deception while maintaining experimental control. It is impossible to find two real-world families affected by flooding that are identical on all dimensions but their skin color and location. While such forms of abstraction do not appear to affect the interpretation of experimental findings [50], our rendering may pose some threat to external validity as it is necessarily a reduction in ecological realism. Second, each vignette specified that the family lived in a “low-income neighborhood” and that the community was “poor” to achieve informational equivalence on this background attribute, to ensure that manipulating race and location does not change respondents’ beliefs about the family’s economic situation and thus its need for assistance [51].

Respondents then evaluated a series of statements about governmental and private aid, as well as the family described in the scenario. The statements remained identical across all four treatment conditions. Support for government aid was measured using the following prompts, with responses standardized and averaged to create an index with a mean of zero and a standard deviation of one:

1. The U.S. government should assist local authorities in helping these families, even if fewer resources are available for other needs in the U.S. [agreement on a 5-point scale]
2. How much aid should the U.S. government allocate to assisting families like this one, calculated in terms of contributions per American taxpayer? [options from 0 to 100 in increments of 10]
3. Let us ask for your opinion about climate aid to communities disproportionately affected by climate change in a slightly different way. Do you think that U.S. spending on climate aid to poor communities like these should increase, decrease or be kept about the same? If you think it should increase or decrease, please specify by how much. [(1) decrease to zero … (7) increase by a huge sum.]

Conservatives, in particular, may oppose diverting governmental resources to foreigners or racial outgroups because of their ideology and yet be willing to *personally* donate to needy foreigners. To ensure that our findings are not confounded by respondents’ views about the appropriate role of the U.S. government in domestic and international redistribution, we also measured willingness to assist privately. We presented respondents with a hypothetical opportunity to donate a portion of their earnings: “Imagine that you received $100 today. You have the option to donate a portion of this money to a charity that helps families like this one. About how much would you personally donate?” [options from 0 to 100 in increments of 10]. We also asked respondents if they were willing to welcome the flood victims into their neighborhoods [agreement on the 5-point scale]. We combine these measures into an index of personal willingness to assist.

Subsequent questions were designed to probe the mechanisms shaping support for cross-national and cross-racial assistance (see summary in Table 1). To capture deservingness, we asked respondents whether potential recipients “must accept some of the blame for” their situation and whether they may “misuse” assistance. Prior work on support for welfare and charitable giving has shown that people who are viewed as responsible for their misfortune [38] or as likely to “cheat” the system [32] are judged less deserving of help. Assessment of blame attribution is particularly suitable because it arises from outgroup attribution bias, widely documented in social psychology literature [17].

**Table 1:** Operationalization of Mechanisms, Manipulation Check, and Placebo Variables.

Variable	Question or statement
Perceived social proximity	How close do you feel to families like this one? [Not at all close, not very close, somewhat close, very close]
Perceived deservingness (index)	1. Communities like this one may misuse public and private assistance. [agreement, 5-point scale] 2. Communities like this one must also accept some of the blame for failing to prepare for extreme storms. [agreement, 5-point scale]
Perceived competence (index) to measure paternalistic views	1. Communities like this one are capable of rebuilding and recovering from storms using their own resources. [agreement on the 5-point scale, reversed in index construction] 2. Communities like this one may be less educated about the dangers of climate change. [agreement on the 5-point scale] 3. What is the best way to assist families like this one, who suffered from a major flood? [Provide food, shelter, or essential services (coded 1) vs. cash compensation (coded 0)]
Emotional attribution (indicator); race, nationality, and socioeconomic status (indicator)	How would you describe the family you just saw in the image? (Select as many as you think apply): Black, White, Asian; scared, brave, angry; poor, middle-class, rich; foreign, American. [coded 1 when selected]

*Note:* Questions to measure victims’ competence and to test the hypothesis about paternalism toward racial outgroups are based on [5]. Race and nationality adjectives are used as a manipulation check. Economic status adjectives are used as a placebo test. Emotional adjectives “scared” and “angry” are used for exploratory hypotheses testing.

We measured paternalistic attitudes by asking respondents to assess the competence of affected communities and their education about climate risks, drawing on research demonstrating that stereotypes of low competence underpin paternalistic prejudice [52]. In addition, we asked about the preferred form of aid, distinguishing between in-kind and cash assistance, building on scholarship showing that paternalistic motivations are associated with support for dependency-oriented, in-kind aid that limits recipients’ agency [5,26].

To address concerns about measurement validity, in addition to reporting results with deservingness and paternalism indices as dependent variables, as we had pre-registered, we also present results separately for each item and report correlations between statements combined in each index in S1 File.

We treat paternalism, deservingness, and social proximity as analytically distinct, though not mutually exclusive, mechanisms that could account for the treatment effect on generosity toward flood victims. Rather than attempting to fully adjudicate among these mechanisms, we estimate treatment effects on measures of each sentiment to assess their plausibility. A null treatment effect suggests that a given mechanism is unlikely to be causal. Identifying the operative mechanism definitively would require additional research with a well-identified mediation design.

Respondents were then asked to describe the family in the image by selecting adjectives from a randomized list of (1) racial and national categories, included as a manipulation check to verify that respondents “received” treatment; (2) the family’s socioeconomic status, included as a placebo test since the vignette stated that the family was poor; and (3) emotions that the family was experiencing, included to assess respondents’ ability to perceive victims’ emotional state. The choice of adjectives was motivated by research that shows that racial prejudice predicts American Whites’ readiness to perceive Black faces as “angry” [53].

Our treatment and main outcome variables were identical across the two studies. Study 2 included several additional questions to understand the findings from Study 1. To understand whether racial prejudice operates in a different way with respect to climate and welfare assistance, we measured support for welfare (in the U.S. condition only) using an index based on the following questions:

1. Should federal funding on welfare for families like this one be increased, decreased, or kept about the same? [coded 1 (decreased a lot) through 5 (increased a lot).]
2. The government in Washington should make every possible effort to improve the social and economic position of families like this one [coded 1 (strongly disagree) through 5 (strongly agree).]

Study 2 also incorporated Baker’s ([5]) Perception of Foreign Poor’s Agency Index, designed to capture common paternalistic stereotypes about foreign aid recipients. The index is based on the following questions:

1. Because of difficult economic circumstances, people in poor countries are unable to help themselves get richer. [coded 1 (strongly disagree) through 5 (strongly agree)]
2. There is little that people in poor countries can do by themselves to improve their livelihoods. [coded 1 (strongly disagree) through 5 (strongly agree)]
3. The only way poor countries could grow richer is with financial help from rich countries. [coded 1 (strongly disagree) through 5 (strongly agree)]

We report summary statistics for the main outcome variables in S1 File. We find considerable variation in responses across respondents, with political party driving much of this variation. The surveys concluded with an identical battery of demographic questions.

## Results

We present our results in terms of the two dimensions of the experimental treatment. We focus the analysis on White respondents because this group remains both numerically and economically dominant in the U.S.—and across much of the Global North—and because prior research has demonstrated that Whites’ racial attitudes and perceptions of social distance exert a particularly strong influence on policy, especially in domains such as redistribution, immigration, and foreign aid [54,55]. Furthermore, much of the literature that underpins our hypotheses is grounded in studies of how White populations respond to racialized cues [e.g., 5, 32, 35]. Although the number of Black and other non-White participants in our sample is too small to support fully powered subgroup analyses, we report exploratory results in S1 File. We also report exploratory mediation analyses in S1 File.

### The effects of victims’ country of residence

**Fig 2:**
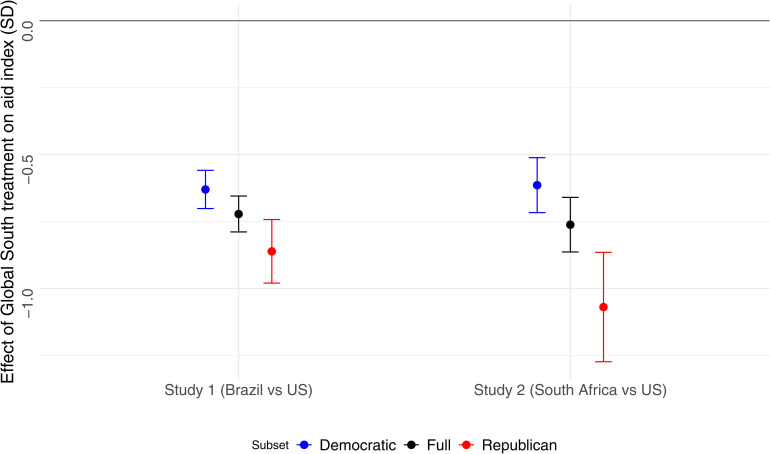
Effect of the Global South treatment on White respondents’ support for governmental aid.

We first examine how flood victims’ country of residence affects (White) respondents’ generosity. In both surveys, we find significantly lower support for government aid toward flood victims living in the Global South relative to those living in the U.S. (see Fig 2). In the full sample, the average recommended government aid per capita stood at $37, and the average personal donation at $27. The Global South treatment reduced support for governmental aid by 0.75 standard deviations (SDs) in each study, which is equivalent to a $22 decrease in per capita aid on a $0–100 scale. This estimate was obtained by multiplying the estimated coefficient on the treatment by the standard deviation ($\sigma_{q}\;$) of responses to the question “How much aid should the U.S. government allocate to assisting families like this one, calculated in terms of contributions per American taxpayer?”, used to calculate the index of support for foreign aid.

The effect of the Global South treatment is negative but smaller in magnitude for personal donations to flood victims, at 0.22 standard deviations for the Brazil treatment (Study 1) and 0.27 standard deviations for the South Africa treatment (Study 2). Respondents are therefore less generous toward foreigners regardless of the nature of assistance or their country of origin.

Various robustness checks are presented in S1 File. For example, the results are virtually identical when covariates (sex, age, education, income, and political party) are included in the regression models. Excluding respondents who did not pass attention checks does not alter our conclusions. We found similar patterns in the subset of African American respondents despite the small sample size.

We are able to confirm that respondents received the location treatment using a follow-up question: only 3.8% of respondents misidentified the family’s nationality across all conditions. The Global South treatment increased probability of selecting “foreign” adjective by 41% (31%) and, correspondingly, decreased the probability of selecting “American” adjective by 55% (57%) in Study 1 (2), as shown in S1 File.

The gap in generosity toward victims in the Global South and the U.S. is largest in the Republican subsample (see Fig 2 and S1 File). The Global South treatment effect is equivalent to a $25 (Brazil) and $31 (South Africa) decrease among Republicans and a $19 (Brazil) and $17 (South Africa) decrease among Democrats in per capita foreign aid on a scale from $0 to $100.

**Fig 3:**
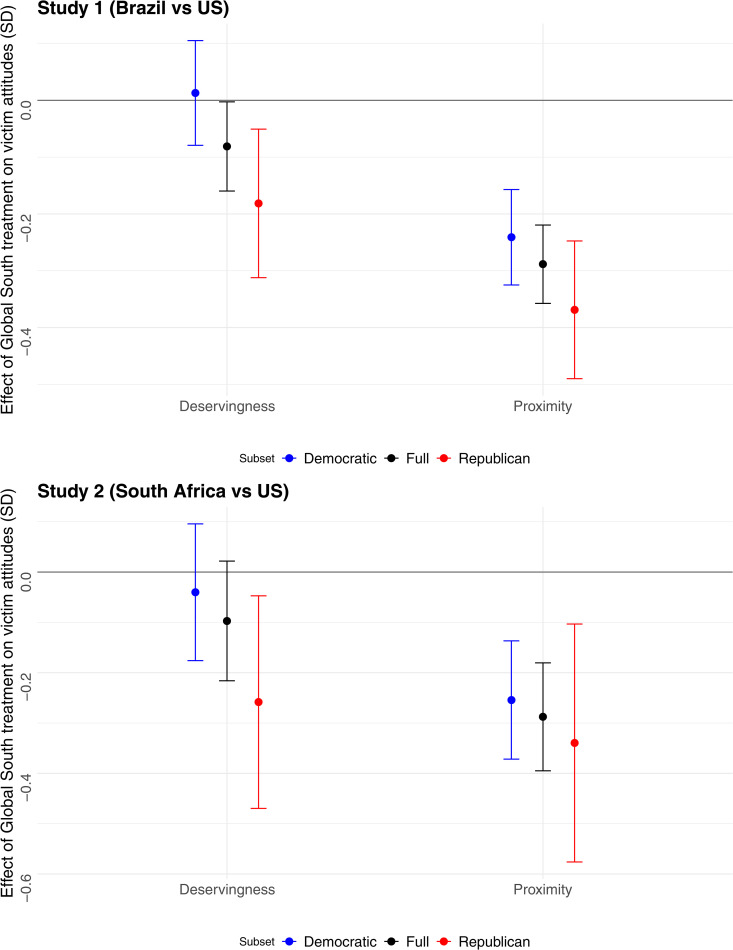
Effect of the Global South treatment on White respondents’ perceptions of victims’ deservingness and social proximity. The top panel presents results from Study 1, which compares victims in Brazil and the U.S. (N = 5,005). The bottom panel presents results from Study 2, which compares victims in South Africa and the U.S. (N = 2,506).

Next, we examine responses to the questions designed to probe which mechanisms are responsible for the gap in generosity toward U.S. and Global South victims. As shown in Fig 3, respondents view foreign victims as less socially proximate, with treatment effects particularly large in the Republican subsample. The effects on social proximity are substantively large, at approximately one-third of a standard deviation in each study. The Global South victims are also perceived as less deserving of assistance, although the effect is statistically significant only among self-identified White Republicans. Breaking the index into its two components for the full sample indicates that the Global South treatment increases respondents’ belief that the receiving community would misuse public and private assistance and that it is to blame for failing to prepare for adverse climate events; however, only the former effect reaches statistical significance (See S1 File). White Republican respondents were 23% (8%) more likely to view South African (Brazilian) flood victims as likely to misuse assistance and 8% (8%) more likely to view South African (Brazilian) victims as responsible for their lack of preparation, relative to US-based flood victims. In both studies, the effects of victims’ nationality on perceived deservingness were null in the White Democrat subsample.

We find no evidence that the Global South treatment reduced respondents’ ability to perceive victims’ emotional state. In fact, respondents were more likely to select the adjective “scared” to characterize victims in the Global South than American victims in (larger) Study 1 (see S1 File). Note that this result should be interpreted with caution because our selection of emotional adjectives was limited.

### The effects of victims’ race

**Fig 4:**
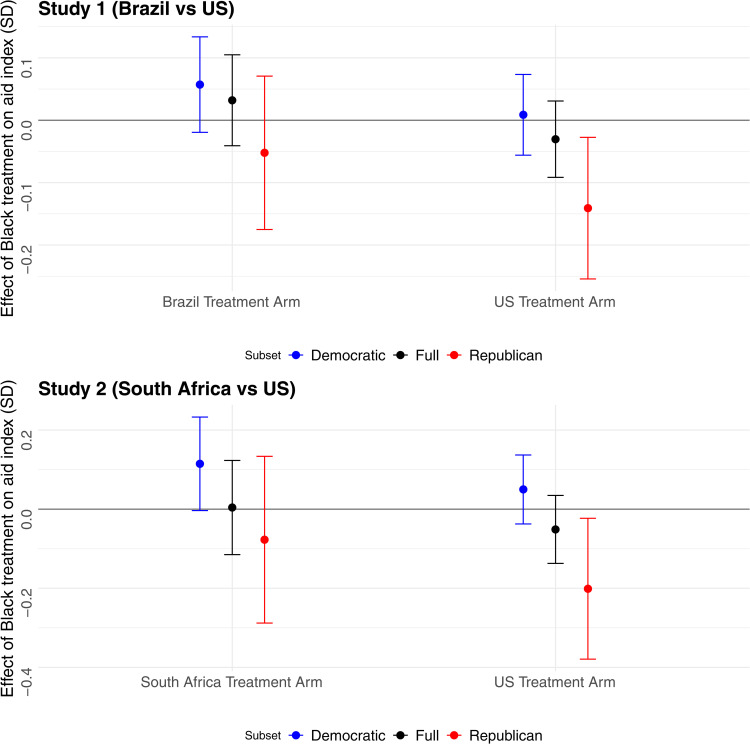
Effect of the race treatment on White respondents’ support for governmental aid. The top panel presents results from Study 1, comparing victims in Brazil and the U.S. (N = 5,005). The bottom panel presents results from Study 2, comparing victims in South Africa and the U.S. (N = 2,506).

Next, we examine how flood victims’ racial identities shape (White) respondents’ support for different forms of aid. In the U.S. condition, the estimated effect of race on support for both government and personal aid is negative but not statistically significant. In the Global South condition, the effect of race approaches zero (see Fig 4 and S1 File). Our expectation of negative racial bias in aid is borne out only in the Republican subsample in the U.S. condition, where the Black treatment has a negative and statistically significant effect on support for governmental aid, equivalent to a $6 decrease in per capita assistance. These patterns suggest that the impact of victims’ race depends on their nationality and that partisanship moderates the effects of race on willingness to assist.

The results are similar when we subset to respondents who passed the attention check, as shown in S1 File. We also find a null effect of race when we ask respondents about welfare attitudes in the U.S., as shown in S1 File. In the smaller subset of Black respondents, the coefficient on Black treatment changes sign and does not reach statistical significance (see S1 File).

**Fig 5:**
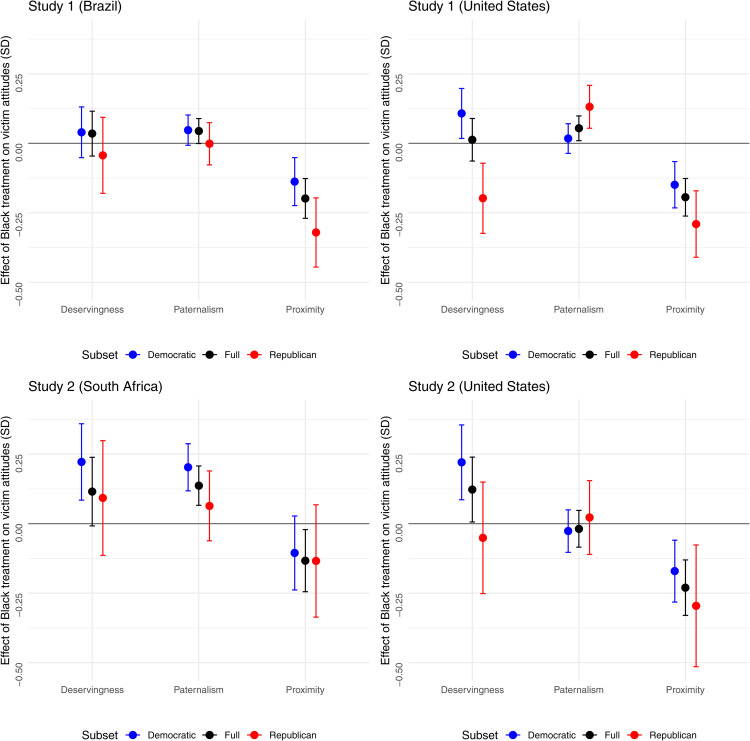
Effect of Black treatment on White respondents’ perceptions of victims’ deservingness, social proximity, and perceived competence. Each panel displays results from a single study and treatment arm.

We further examined whether the race treatment influenced (White) respondents’ perceptions of the victims’ social proximity, deservingness, and competence (Fig 5). We found that Black victims were consistently perceived as less socially proximate than White victims, with an effect size equivalent to 0.3 SDs. The estimates were largest for Republicans in the U.S. and Brazil conditions. In the South Africa condition, partisan differences were negligible.

On the other hand, the estimated effects of race on perceptions of victims’ deservingness and competence (paternalism channel) were significant only in some treatment conditions and subsamples. The null effect of race on deservingness in the full sample masked important partisan differences. Self-identified Democrats were more likely to view Black victims as deserving of aid compared to White victims, with statistically significant effects in the U.S. and South African conditions. Notably, White Democrats were 25% less likely than White Republicans to agree that Black communities “must also accept some of the blame for failing to prepare for extreme storms.” By contrast, Republicans were significantly less inclined to state that Black American victims were deserving of aid (compared to White American victims). No differences in perceived deservingness by victims’ race emerged in the Global South condition.

We find suggestive evidence of greater paternalism toward Black victims in the Global South: Respondents were more likely to agree that Black communities were less “educated about the dangers of climate change” and less “capable of rebuilding and recovering from storms using their own resources” than White communities, but the coefficient reached statistical significance only in the South Africa condition (Study 2). This treatment effect was larger among Democrats.

In the (larger) Study 1, we also found that respondents were more likely to perceive Black communities in the U.S. as less educated and capable than White communities, with the effect larger among Republicans.

Results from Study 2, which also included Baker’s ([5]) Perception of the Foreign Poor’s Agency Index, further show that when presented with Black Global South victims, both Republicans and Democrats were more likely to express paternalistic views of the foreign poor, with the effect significant at 5% level for Democrats and 10% level for Republicans (see S1 File). This pattern is consistent with Baker’s ([5]) argument that White Americans perceive citizens of Black-majority countries as “in need of paternalistic saving.”

## Discussion

While 65 percent of Americans say they support disaster assistance to foreign countries [56], our findings show the very strong extent to which they are more generous to co-nationals as compared with foreign victims, irrespective of the victims’ race. An experimentally-assigned Global South treatment reduced support for U.S. government aid by 0.75 SDs—or $22 on a $0–$100 scale—in both Brazil and South Africa conditions. Willingness to personally assist was also 0.22–0.27 SDs lower.

Partisanship emerged as a strong predictor across all forms of assistance: Republicans were less supportive of both public and private aid, suggesting the pattern goes beyond disagreement over government responsibility.

The Global South treatment increased perceived social distance between respondents and disaster victims. Among Republicans, it also reduced victims’ perceived deservingness: Republicans were more likely to believe that victims would misuse aid or were to blame for failing to prepare for extreme weather when victims were based in the Global South. This pattern aligns with research showing that support for international aid varies by political ideology [10,46].

In our study, we found that race cues mattered less than national cues and were moderated by partisanship, which we explain as a function of Republicans being more likely to hold a view of nationhood that is more restrictive in its criteria for membership. Specifically, in the U.S. condition, the coefficient on race was negative in the full sample and statistically significant among Republicans. In the Global South, the null result in the full sample masked heterogeneity by party: Democrats were slightly *more likely* to assist Black victims than White victims, while Republicans were *less likely* to assist Black victims than White victims. These results did not reach statistical significance. Across both U.S. and Global South conditions, (White) respondents perceived Black victims as less socially proximate than White victims, by 0.12–0.25 SDs, although the effect of race on social proximity was smaller than the effect of nationality (0.35–0.37 SDs). Race had divergent effects on U.S. victims’ perceived deservingness among Democrats and Republicans: Democrats were less likely to blame racial outgroups for failing to prepare and/or to expect them to misuse aid, while Republicans were more likely to do so.

We found mixed support for the paternalism channel proposed by [5]. White Democrats demonstrated more paternalistic views toward Black victims than White victims in the South Africa condition, but not in the Brazil condition. For White Republicans, the effects of race on paternalism were significant only in the U.S. condition in (larger) Study 1. Counterintuitively, this subgroup of respondents perceived Black US-based victims as both less competent and more to blame for their plight than White US-based victims.

While climate and disaster aid are not as “race-coded” as welfare policy in the U.S., we find similar results on respondents’ support for welfare, with the coefficient on Black victims treatment negative and statistically significant for Republicans. These findings replicate results in other work [35–38] that signals about race *alone* may be insufficient to explain the gap in charitable behavior and/or support for redistribution. Instead, our analysis indicates that partisanship moderates the effects of victim’s race on willingness to assist: only specific subgroups of the U.S. population will discriminate against racial outgroups when they are in obvious need.

Like [3], who find Americans less supportive of FEMA aid to Puerto Rican than Houston-based hurricane victims, we show that victims’ foreignness reduces generosity. [3] attribute this to “attitudes about the place and the people who live there.” Our findings also align with [2], who finds that dark skin treatment has a more negative impact on Americans’ attitudes toward English- than Spanish-speaking Puerto Ricans – a pattern she attributes to the latter’s perceived “foreignness.” We extend this work by testing mechanisms behind racial and national biases in aid, finding that victims’ perceived deservingness—often assumed but rarely measured—cannot fully account for giving gaps. Instead, we highlight the role of social distance and perceived competence in shaping aid preferences.

In the larger sample, our results suggest that the deservingness heuristic–central to support for domestic redistribution–may be less applicable to disaster aid, where nature, rather than human decisions are presumed to be at fault [57]. However, Republicans were more likely to perceive racial outgroups as responsible for their misfortune. Political ideology thus shapes not just climate aid preferences [11,58], but also how responsibility is assigned.

We note some limitations of our study with respect to external validity. First, our treatment is based on an artist’s rendering of a flood, and real-world exposure to events in the form of photographic and video coverage may have different effects. Relatedly, we consider just two Global South countries, and citizens might perceive deservingness differently in other locations. Finally, while broadly comparable to national benchmarks, our sample is modestly more liberal and more highly educated than the U.S. population.

Future research should further examine key theoretical questions raised here, including whether—and under what conditions—perceptions of low competence elicit generosity rather than contempt. Relatedly, future work should explore how and why partisan attachments shape responses to external stimuli, including through mechanisms such as nationalism and racial resentment. Attention to these processes may help explain why issues like climate change and foreign assistance have become so deeply polarized.

## Supporting information

S1 File Supplementary tables and figures. (PDF)

## References

[1] Gilens M, Mendelberg T, Short N. Racial Attitudes and Views of Disaster. Political Research Quarterly. 2024;78(2):468–80. doi: 10.1177/10659129241306762

[2] RIVERA-BURGOS V. Language, Skin Tone, and Attitudes toward Puerto Rico in the Aftermath of Hurricane Maria. Am Polit Sci Rev. 2022;117(3):789–804. doi: 10.1017/s0003055422000971

[3] Ross AD, Rouse SM, Alcañiz I, Marchevsky A. Who Is Perceived as Deserving? How Social Identities Shape Attitudes about Disaster Assistance in the United States. APSC. 2024;57(4):521–8. doi: 10.1017/s1049096524000362

[4] Freeman B, Kim DG, Lake DA. Race in International Relations: Beyond the “Norm Against Noticing”. Annu Rev Polit Sci. 2022;25(1):175–96. doi: 10.1146/annurev-polisci-051820-120746

[5] Baker A. Race, paternalism, and foreign aid: Evidence from U.S. public opinion. American Political Science Review. 2015;109(1):93–109. doi: 10.1017/S0003055414000549

[6] Simon CA, Moltz MC. Immigrant Citizens and Racial Resentment in International Policy Perspective: The Role of Nativity and Racial Resentment in Shaping Support for U.S. Foreign Assistance Expenditure, 2002–2016. Development. 2019;62(1–4):186–95. doi: 10.1057/s41301-019-00225-0

[7] Dworkin G. Paternalism. The Monist. 1972;56(1):64–84. doi: 10.5840/monist197256119

[8] Bonikowski B, Feinstein Y, Bock S. The Partisan Sorting of “America”: How Nationalist Cleavages Shaped the 2016 U.S. Presidential Election. American Journal of Sociology. 2021;127(2):492–561. doi: 10.1086/717103

[9] Enders AM. A Matter of Principle? On the Relationship Between Racial Resentment and Ideology. Polit Behav. 2019;43(2):561–84. doi: 10.1007/s11109-019-09561-w

[10] Milner HV, Tingley D. Public Opinion and Foreign Aid: A Review Essay. International Interactions. 2013;39(3):389–401. doi: 10.1080/03050629.2013.784090

[11] Ansah PO, Campbell E, Kotcher J, Rosenthal SA, Leiserowitz A, Maibach E. Predictors of U.S. public support for climate aid to developing countries. Environ Res Commun. 2023;5(12):125003. doi: 10.1088/2515-7620/ad0ff2

[12] Bloom PA. Against empathy: The case for rational compassion. New York: Random House. 2016.

[13] Singer P. Famine, affluence, and morality. Oxford: Oxford University Press. 2015.

[14] James TK, Zagefka H. The effects of group memberships of victims and perpetrators in humanly caused disasters on charitable donations to victims. J Applied Social Pyschol. 2017;47(8):446–58. doi: 10.1111/jasp.12452

[15] Levine M, Thompson K. Identity, place, and bystander intervention: social categories and helping after natural disasters. J Soc Psychol. 2004;144(3):229–45. doi: 10.3200/SOCP.144.3.229-245 15168427

[16] Heermann M, Koos S, Leuffen D. Who Deserves European Solidarity? How Recipient Characteristics Shaped Public Support for International Medical and Financial Aid during COVID-19. Brit J Polit Sci. 2022;53(2):629–51. doi: 10.1017/s0007123422000357

[17] Pettigrew TF. Intergroup attribution. Oxford Research Encyclopedia of Psychology. 2020. 10.1093/acrefore/9780190236557.013.326

[18] Turner JC, Hogg MA, Oakes PJ, Reicher SD, Wetherell MS. Rediscovering the social group: A self-categorization theory. Oxford: Blackwell. 1987.

[19] Cuddy AJC, Rock MS, Norton MI. Aid in the Aftermath of Hurricane Katrina: Inferences of Secondary Emotions and Intergroup Helping. Group Processes & Intergroup Relations. 2007;10(1):107–18. doi: 10.1177/1368430207071344

[20] Riva P, Andrighetto L. “Everybody feels a broken bone, but only we can feel a broken heart”: Group membership influences the perception of targets’ suffering. Euro J Social Psych. 2012;42(7):801–6. doi: 10.1002/ejsp.1918

[21] Cikara M, Farnsworth RA, Harris LT, Fiske ST. On the wrong side of the trolley track: neural correlates of relative social valuation. Soc Cogn Affect Neurosci. 2010;5(4):404–13. doi: 10.1093/scan/nsq011 20150342 PMC2999760

[22] Harris LT, Fiske ST. Dehumanizing the lowest of the low: neuroimaging responses to extreme out-groups. Psychol Sci. 2006;17(10):847–53. doi: 10.1111/j.1467-9280.2006.01793.x 17100784

[23] Tan Q, Huang Y, Ling Z, Zhan Y, Zhou H. Warmer Individuals Get More Help: The Influence of Stereotypes and Empathy on Moral Decision-Making. Psychol Rep. 2024;127(6):2980–98. doi: 10.1177/00332941231152386 36680548

[24] Becker JC, Asbrock F. What triggers helping versus harming of ambivalent groups? Effects of the relative salience of warmth versus competence. Journal of Experimental Social Psychology. 2012;48(1):19–27. doi: 10.1016/j.jesp.2011.06.015

[25] Nadler A. Inter-group helping relations as power relations: Helping relations as affirming or challenging inter-group hierarchy. Journal of Social Issues. 2002;58:487–503. doi: 10.1111/1540-4560.00272

[26] Halabi S, Dovidio JF, Nadler A. When and How Do High Status Group Members Offer Help: Effects of Social Dominance Orientation and Status Threat. Political Psychology. 2008;29(6):841–58. doi: 10.1111/j.1467-9221.2008.00669.x

[27] Schroeder J, Waytz A, Epley N. Endorsing help for others that you oppose for yourself: Mind perception alters the perceived effectiveness of paternalism. J Exp Psychol Gen. 2017;146(8):1106–25. doi: 10.1037/xge0000320 28557510

[28] Cuddy AJC, Fiske ST, Glick P. Warmth and Competence as Universal Dimensions of Social Perception: The Stereotype Content Model and the BIAS Map. Advances in Experimental Social Psychology. Elsevier. 2008. p. 61–149. 10.1016/s0065-2601(07)00002-0

[29] Fiske ST, Cuddy AJC, Glick P, Xu J. A model of (often mixed) stereotype content: competence and warmth respectively follow from perceived status and competition. J Pers Soc Psychol. 2002;82(6):878–902. doi: 10.1037/0022-3514.82.6.878 12051578

[30] Schofield TP, Suomi A, Butterworth P. Is the stereotype of welfare recipients associated with type of welfare state regime? A cross‐national meta‐regression of the stereotype content model. J Applied Social Psychol. 2021;52(4):201–9. doi: 10.1111/jasp.12843

[31] Berry L. Americans prioritize domestic spending over foreign aid, 2024. Public Opinion Survey Report. https://globalaffairs.org/research/public-opinion-survey/americans-prioritize-domestic-spending-over-foreign-aid

[32] Gilens M. Why Americans hate welfare: Race, media, and the politics of antipoverty policy. Chicago: University of Chicago Press. 1999.

[33] Winter JG. Dangerous frames: How ideas about race and gender shape public opinion. Chicago: University of Chicago Press. 2008.

[34] Hancock A-M. The politics of disgust: The public identity of the welfare queen. NYU Press, New York, 2004.

[35] Huddy L, Feldman S. On Assessing the Political Effects of Racial Prejudice. Annu Rev Polit Sci. 2009;12(1):423–47. doi: 10.1146/annurev.polisci.11.062906.070752

[36] Fong CM, Luttmer EFP. What Determines Giving to Hurricane Katrina Victims? Experimental Evidence on Racial Group Loyalty. American Economic Journal: Applied Economics. 2009;1(2):64–87. doi: 10.1257/app.1.2.64

[37] Fong CM, Luttmer EFP. Do fairness and race matter in generosity? Evidence from a nationally representative charity experiment. Journal of Public Economics. 2011;95(5–6):372–94. doi: 10.1016/j.jpubeco.2010.07.010

[38] Gross K, Wronski J. Helping the Homeless: The Role of Empathy, Race and Deservingness in Motivating Policy Support and Charitable Giving. Polit Behav. 2019;43(2):585–613. doi: 10.1007/s11109-019-09562-9

[39] Adida CL, Lo A, Platas MR. Americans preferred Syrian refugees who are female, English-speaking, and Christian on the eve of Donald Trump’s election. PLoS One. 2019;14(10):e0222504. doi: 10.1371/journal.pone.0222504 31600224 PMC6786519

[40] Bansak K, Hainmueller J, Hangartner D. How economic, humanitarian, and religious concerns shape European attitudes toward asylum seekers. Science. 2016;354(6309):217–22. doi: 10.1126/science.aag2147 27708060

[41] Bansak K, Hainmueller J, Hangartner D. Europeans’ support for refugees of varying background is stable over time. Nature. 2023;620(7975):849–54. doi: 10.1038/s41586-023-06417-6 37558879 PMC10447233

[42] Hopkins DJ, Washington S. The rise of Trump, the fall of prejudice? Tracking white Americans’ racial attitudes via a panel survey, 2008–2018. Public Opinion Quarterly. 2020;84(1):119–40. doi: 10.1093/poq/nfaa004

[43] Stern C, Axt J. Ideological Differences in Race and Gender Stereotyping. Social Cognition. 2021;39(2):259–94. doi: 10.1521/soco.2021.39.2.259

[44] Sniderman PM, Crosby GC, Howell WG. The politics of race. In: Sears DO, Sidanius J, Bobo L, editors. Racialized politics: The debate about racism in America. Princeton, NJ: University of Chicago Press. 2000.

[45] Goenka S, Thomas M. Are Conservatives Less Likely Than Liberals to Accept Welfare? The Psychology of Welfare Politics. Journal of the Association for Consumer Research. 2022;7(3):305–15. doi: 10.1086/719586

[46] Greene ZD, Licht AA. Domestic Politics and Changes in Foreign Aid Allocation: The Role of Party Preferences. Political Research Quarterly. 2017;71(2):284–301. doi: 10.1177/1065912917735176

[47] Douglas D, Ewell PJ, Brauer M. Data quality in online human-subjects research: Comparisons between MTurk, Prolific, CloudResearch, Qualtrics, and SONA. PLOS ONE. 2023;180(3):0 e0279720. doi: 10.1371/journal.pone.0279720 PMC10013894 36917576

[48] Scott R. Does university make you more liberal? Estimating the within-individual effects of higher education on political values. Electoral Studies. 2022;77:102471. doi: 10.1016/j.electstud.2022.102471

[49] Marx AN. Making race and nation: A comparison of South Africa, the United States, and Brazil. Cambridge: Cambridge University Press. 1997. 10.1017/CBO9780511810480

[50] Brutger R, Kertzer JD, Renshon J, Tingley D, Weiss CM. Abstraction and Detail in Experimental Design. American J Political Sci. 2022;67(4):979–95. doi: 10.1111/ajps.12710

[51] Dafoe A, Zhang B, Caughey D. Information Equivalence in Survey Experiments. Polit Anal. 2018;26(4):399–416. doi: 10.1017/pan.2018.9

[52] Susan T. Fiske and Michael S. North. Chapter 24 - Measures of stereotyping and prejudice: Barometers of bias. In Gregory J. Boyle, Donald H. Saklofske, and Gerald Matthews, editors, Measures of Personality and Social Psychological Constructs, pages 684–718. Academic Press, San Diego, 2015. 10.1016/B978-0-12-386915-9.00024-3

[53] Hugenberg K, Bodenhausen GV. Facing prejudice: implicit prejudice and the perception of facial threat. Psychol Sci. 2003;14(6):640–3. doi: 10.1046/j.0956-7976.2003.psci_1478.x 14629699

[54] Kinder DR, Sanders LM. Divided by color: racial politics and democratic ideals. Chicago, IL: University of Chicago Press. 1996.

[55] Jardina A. White identity politics. Cambridge: Cambridge University Press. 2019.

[56] Montgomery D. Americans support foreign disaster aid, but split over climate change’s role in disasters, 2023. https://today.yougov.com/politics/articles/47370-poll-foreign-disaster-aid-climate-change

[57] Zagefka H, Noor M, Brown R, de Moura GR, Hopthrow T. Donating to disaster victims: Responses to natural and humanly caused events. Eur J Soc Psychol. 2010;41(3):353–63. 10.1002/ejsp.781

[58] Egan PJ, Mullin M. Climate Change: U.S. Public Opinion. Annu Rev Polit Sci. 2017;20(1):209–27. 10.1146/annurev-polisci-051215-022857

