# Supplementary material for: Race, nationality, and partisanship shape U.S. public support for climate disaster aid: Evidence from two survey experiments
Source: PLoS One. 2026 May 6;21(5):e0347292. doi: 10.1371/journal.pone.0347292 (PMC13148718; doi:10.1371/journal.pone.0347292)
Supplement: S1 File — (PDF) [file pone.0347292.s001.pdf]

S1 File: Supplementary tables and figures for 'Nationality, and Partisanship Shape U.S. Public Support for Climate Disaster Aid: Evidence from Two Survey Experiments'

Contents

A Descriptive statistics about the Prolific sample **S1**

B Descriptive statistics on main outcome variables **S2**

C Supplementary Results **S3**
C.1 Main results: Global South treatment . . . S3
C.2 Mediation analysis: Global South treatment . . . S4
C.3 Main results: Race treatment . . . S7
C.4 Emotional attribution . . . S8
C.5 Mediation analysis: Race treatment . . . S9
C.6 Results by individual attitudes . . . S11
C.7 Main results: Race treatment . . . S12

D Robustness checks **S13**

E Additional results **S15**

A Descriptive statistics about the Prolific sample

Table A1. Summary statistics of the survey sample compared against the US population.

	Study 1	Study 2	US Population
Age	41.93	39.26	38.8
Female	0.52	0.51	0.51
White	0.76	0.64	0.75
Black	0.11	0.17	0.14
Hispanic	0.09	0.11	0.20
High-school degree	0.87	0.89	0.89
College degree	0.66	0.69	0.34
California	0.09	0.13	0.12
Texas	0.07	0.08	0.09
Democrat	0.50	0.53	0.35
Independent, leaning Democrat	0.18	0.18	0.11
Independent, leaning Republican	0.10	0.11	0.11
Republican	0.19	0.17	0.32
Annual family income above $100k	0.25	0.30	0.31
Annual family income under $20k	0.10	0.08	0.14
Believe global warming is real	0.84	0.87	0.73
Believe global warming will affect them	0.74	0.76	0.43
No travel outside US	0.56	0.51	–
Sample size	5,005	2,506	Varies

Sources for data on the US population: US census (population demographics in 2020); American National Election Studies (partisanship in 2020); American Community Survey (household income in 2020); Yale Program on Climate Change Communication (beliefs about global warming in 2020). All values are sample means.

B Descriptive statistics on main outcome variables

Table A2. Summary statistics for main outcome variables.

	Mean					
	Study 1 (1)	Study 2 (2)	White (3)	Democrat (4)	Republican (5)	Pooled (6)
USG should assist	3.66	3.62	3.61	3.84	3.13	3.64
USG per capita aid	39.97	40.07	38.09	41.64	30.39	40.01
USG increase/decrease aid	4.76	4.74	4.68	5.18	3.64	4.76
Willing to host family	3.92	3.92	3.95	4.18	3.45	3.92
Donate to family	31.27	32.17	29.83	31.28	26.66	31.56
Misuse assistance	2.47	2.46	2.50	2.25	3.04	2.47
Blame family	2.51	2.46	2.52	2.28	3.04	2.49
Family is less capable	3.61	3.59	3.62	3.80	3.22	3.60
Family is less educated	3.45	3.37	3.44	3.55	3.22	3.42
Prefer in-kind aid	0.86	0.87	0.87	0.84	0.92	0.86
Feel close to family	2.52	2.47	2.50	2.57	2.35	2.50
Support for gov. aid index	0.00	0.00	-0.05	0.17	-0.50	0.00
Personal aid index	-0.01	0.00	-0.02	0.10	-0.28	-0.00
Paternalism index	0.29	0.29	0.30	0.38	0.14	0.29
Deservingness index	0.01	-0.00	-0.02	0.19	-0.46	0
Feel close to family	2.50	2.52	2.47	2.50	2.57	2.35
Support for gov. aid index	0.00	0.00	0.00	-0.05	0.17	-0.50
Personal aid index	-0.00	-0.01	0.00	-0.02	0.10	-0.28
Paternalism index	0.29	0.29	0.29	0.30	0.38	0.14
Deservingness index	0.00	0.01	-0.00	-0.02	0.19	-0.46
Foreign poor agency index		0.00	-0.04	0.16	-0.48	0.00
US welfare index		0.00	-0.05	0.25	-0.80	0.00

Columns 1 and 2 present sample means for Study 1 and 2, respectively. Columns 3-6 present sample means for data pooled from both studies, including for subsets of White, Democrat, and Republican respondents.

C Supplementary Results

C.1 Main results: Global South treatment

Table A3. Effect of Global South treatment on support for government aid and willingness to personally aid victims.

	Panel A. DV: Support for governmental aid							
	Study 1				Study 2			
	White (1)	White (2)	Dem. (3)	Rep. (4)	White (5)	White (6)	Dem. (7)	Rep. (8)
Brazil	-0.73^{***}	-0.72^{***}	-0.65^{***}	-0.83^{***}				
	(0.03)	(0.03)	(0.04)	(0.06)				
	[0.00]	[0.00]	[0.00]	[0.00]				
South Africa					-0.75^{***}	-0.76^{***}	-0.61^{***}	-1.07^{***}
					(0.05)	(0.05)	(0.05)	(0.10)
					[0.00]	[0.00]	[0.00]	[0.00]
Male		-0.12^{***}	-0.06^{*}	0.09		-0.09^{*}	-0.05	-0.02
		(0.03)	(0.04)	(0.06)		(0.05)	(0.05)	(0.10)
Income		-0.03^{***}	-0.01	-0.02		-0.03^{**}	-0.03^{***}	0.00
		(0.01)	(0.01)	(0.02)		(0.01)	(0.01)	(0.02)
College		-0.01	-0.02	-0.11^{*}		0.09	0.01	0.06
		(0.04)	(0.04)	(0.07)		(0.06)	(0.06)	(0.11)
No travel		-0.01	0.04	-0.06		-0.01	-0.08	0.30^{***}
		(0.04)	(0.04)	(0.06)		(0.06)	(0.06)	(0.11)
R^2	0.20	0.21	0.23	0.25	0.22	0.24	0.23	0.35
Num. obs.	1959	1913	1225	649	773	754	519	211
	Panel B. DV: Willingness to personally assist							
	Study 1				Study 2			
	White (1)	White (2)	Dem. (3)	Rep. (4)	White (5)	White (6)	Dem. (7)	Rep. (8)
Brazil	-0.22^{***}	-0.22^{***}	-0.14^{***}	-0.34^{***}				
	(0.03)	(0.03)	(0.04)	(0.06)				
	[0.00]	[0.00]	[0.00]	[0.00]				
South Africa					-0.28^{***}	-0.27^{***}	-0.13^{**}	-0.54^{***}
					(0.05)	(0.05)	(0.06)	(0.11)
					[0.00]	[0.00]	[0.00]	[0.00]
Male		-0.18^{***}	-0.15^{***}	-0.16^{***}		-0.19^{***}	-0.17^{***}	-0.15
		(0.03)	(0.04)	(0.06)		(0.05)	(0.06)	(0.11)
Income scale		0.00	0.03^{***}	-0.02		-0.02^{*}	-0.03^{*}	0.00
		(0.01)	(0.01)	(0.02)		(0.01)	(0.01)	(0.03)
College		-0.01	0.03	-0.13^{**}		0.02	0.00	-0.02
		(0.04)	(0.04)	(0.07)		(0.06)	(0.07)	(0.12)
No int'l travel		0.04	0.09^{**}	-0.01		-0.02	-0.06	0.16
		(0.04)	(0.04)	(0.07)		(0.06)	(0.06)	(0.12)
R^2	0.02	0.04	0.03	0.08	0.03	0.05	0.03	0.12
Num. obs.	1960	1914	1225	650	773	754	519	211

Notes: Study 1 compared flood victims in the US and Brazil. Study 2 compared flood victims in the US and South Africa. Columns 1-2 and 5-6 use full sample of White respondents. Columns 3-4 and 7-8 subset to self-identified White Democrats and Republicans. Intercept is omitted from the table. Heteroskedasticity-robust standard errors in parentheses. P-values obtained using randomization inference are in square brackets. $^{***}p < 0.001$; $^{**}p < 0.01$; $^{*}p < 0.05$

C.2 Mediation analysis: Global South treatment

Within the context of a vignette experiment, it is extremely difficult to adjudicate among causal mechanisms between the assignment of treatment and studied outcomes. We report on some exploratory mediation analyses here with respect to the Global South treatment and below with respect to the race treatment.

Specifically, we perform mediation analysis to estimate how much of the effect of the Global South treatment on support for government aid operates via the hypothesized mechanisms of social proximity and deservingness [1]. This analysis was not registered in our pre-analysis plan, which specified which questions would be used to measure mechanisms (mediators) but did not specify using formal mediation estimation. In brief, mediation analysis breaks down the effect of the treatment on the outcome into two parts: the mediated effect of the treatment on the outcome through the mediator (ACME) and the unmediated effect of the treatment on the outcome, i.e. the remaining effect unexplained by the mediator (average direct effect, or ADE). The sum of the ADE and ACME is equal to the total treatment effect.

Mediation analysis relies on the (untestable) sequential ignorability assumption, which requires that the mediator is independent of unobserved confounders, conditional on treatment status and pre-treatment covariates. Since we did not experimentally manipulate deservingness or social proximity, we cannot be sure that our estimates are unbiased. Nonetheless, the mediation analysis provides some insights into the relationships between the observed Global South treatment effect and respondents' perceptions of the flood victims.

The results in Table A4 confirm that victims' perceived proximity and deservingness predict both governmental and private aid. Table A4 further shows that the ACME of social proximity is statistically significant in both studies and nearly three times as large as the ACME of deservingness, which is significant only in Study 1. In Table A5, we replicate these findings for the White Republican sample and find that the ACMEs are directionally consistent with the full White sample and statistically significant for *both* deservingness and social proximity. In this sample, the ACME of deservingness amounts to 42% (Study 1) and 75% (Study 2) of the ACME of social proximity. The deservingness mechanism thus appears to play a more important role in mediating the effects of the Global South treatment among Republicans than among Democrats. However, both social proximity and deservingness account for only a small proportion of the total effect of nationality (i.e., the ACME is considerably smaller than the ADE), indicating that additional causal mechanisms may be influencing the outcome.

Table A4. Mediation analysis for the effect of the Global South treatment.

	Deservingness				Proximity			
	Study 1		Study 2		Study 1		Study 2	
	(1)	(2)	(1)	(2)	(1)	(2)	(1)	(2)
Global South Treatment	-0.69***	-0.10**	-0.73***	-0.10	-0.63***	-0.39***	-0.68***	-0.36***
	(0.03)	(0.04)	(0.05)	(0.06)	(0.03)	(0.04)	(0.05)	(0.07)
Mechanism	0.35***		0.31***		0.23***		0.23***	
	(0.02)		(0.03)		(0.02)		(0.03)	
ACME	-0.03**		-0.03		-0.09***		-0.08***	
	[-0.07,-0.01]		[-0.06,0.01]		[-0.12,-0.07]		[-0.12,-0.05]	
ADE	-0.68***		-0.74***		-0.63***		-0.68***	
	[-0.77,-0.62]		[-0.81,-0.65]		[-0.71,-0.57]		[-0.77,-0.6]	
R^2	0.34	0.03	0.34	0.07	0.28	0.06	0.31	0.05
Num. obs.	1913	1913	754	754	1912	1912	754	754

Notes: Study 1 compared flood victims in the US and Brazil. Study 2 compared flood victims in the US and South Africa. Columns marked (1) refer to the outcome model and contain the coefficients of the treatment and the mediator variables on the outcome variable. Columns marked (2) refer to the mediator model and contain the coefficient of the treatment on the mediator variable. $^{***}p < 0.001$; $^{**}p < 0.01$; $^{*}p < 0.05$

Table A5. Mediation analysis for the effect of the Global South treatment, White Republican subsample.

	Deservingness				Proximity			
	Study 1		Study 2		Study 1		Study 2	
	(1)	(2)	(1)	(2)	(1)	(2)	(1)	(2)
Treatment	-0.77***	-0.16**	-0.98***	-0.26**	-0.71***	-0.47***	-0.95***	-0.42***
	(0.06)	(0.07)	(0.10)	(0.11)	(0.06)	(0.08)	(0.10)	(0.15)
Mechanism	0.34***		0.36***		0.25***		0.29***	
	(0.03)		(0.06)		(0.03)		(0.04)	
ACME	-0.05**		-0.09***		-0.12***		-0.12***	
	[-0.09,-0.01]		[-0.17,-0.02]		[-0.16,-0.08]		[-0.22,-0.04]	
ADE	-0.77***		-0.98***		-0.71***		-0.95***	
	[-0.89,-0.68]		[-1.16,-0.81]		[-0.81,-0.58]		[-1.12,-0.77]	
R^2	0.35	0.02	0.44	0.08	0.32	0.10	0.46	0.08
Num. obs.	649	649	211	211	649	649	211	211

Notes: Study 1 compared flood victims in the US and Brazil. Study 2 compared flood victims in the US and South Africa. Columns marked (1) refer to the outcome model and contain the coefficients of the treatment and the mediator variables on the outcome variable. Columns marked (2) refer to the mediator model and contain the coefficient of the treatment on the mediator variable. $^{***}p < 0.001$; $^{**}p < 0.01$; $^{*}p < 0.05$

C.3 Main results: Race treatment

Table A6. Effect of race treatment on support for government aid

	Panel A: Global South arm							
	Brazil (Study 1)				South Africa (Study 2)			
	White (1)	White (2)	Dem. (3)	Rep. (4)	White (5)	White (6)	Dem. (7)	Rep. (8)
Black Treatment	0.03	0.02	0.06	−0.06	−0.01	0.01	0.11	−0.08
	(0.04)	(0.04)	(0.04)	(0.06)	(0.06)	(0.06)	(0.06)	(0.11)
RI	[0.45]	[0.58]	[0.13]	[0.34]	[0.91]	[0.93]	[0.06]	[0.46]
Male		-0.12^{**}	−0.02	−0.06		−0.11	0.03	−0.11
		(0.04)	(0.04)	(0.06)		(0.06)	(0.06)	(0.10)
Income		-0.03^{**}	−0.01	−0.03		−0.02	-0.03^{*}	0.04
		(0.01)	(0.01)	(0.02)		(0.01)	(0.01)	(0.02)
College		0.05	−0.02	0.01		0.02	0.03	−0.21
		(0.04)	(0.04)	(0.07)		(0.07)	(0.07)	(0.12)
No travel		−0.06	0.03	−0.12		-0.16^{*}	−0.09	−0.12
		(0.04)	(0.04)	(0.07)		(0.06)	(0.07)	(0.11)
R^2	0.00	0.01	0.00	0.01	0.00	0.01	0.02	0.03
Num. obs.	1924	1892	1217	637	777	765	497	250

	Panel B: US arm							
	US (Study 1)				US (Study 2)			
	White (1)	White (2)	Dem. (3)	Rep. (4)	White (5)	White (6)	Dem. (7)	Rep. (8)
Black Treatment	−0.04	−0.04	−0.02	−0.11	−0.05	−0.05	0.05	-0.20^{*}
	(0.03)	(0.03)	(0.03)	(0.06)	(0.04)	(0.04)	(0.04)	(0.09)
	[0.18]	[0.25]	[0.52]	[0.07]	[0.30]	[0.24]	[0.28]	[0.03]
Male		-0.08^{*}	−0.01	−0.09		-0.12^{**}	−0.07	−0.10
		(0.03)	(0.03)	(0.06)		(0.04)	(0.04)	(0.09)
Income		-0.02^{**}	−0.01	-0.03^{*}		-0.04^{***}	−0.02	−0.04
		(0.01)	(0.01)	(0.01)		(0.01)	(0.01)	(0.02)
College		0.01	−0.04	−0.01		0.10	−0.02	0.23^{*}
		(0.03)	(0.04)	(0.06)		(0.05)	(0.05)	(0.10)
No travel		−0.04	−0.07	0.04		−0.04	−0.04	0.13
		(0.03)	(0.04)	(0.06)		(0.05)	(0.05)	(0.10)
R^2	0.00	0.01	0.01	0.03	0.00	0.03	0.02	0.07
Num. obs.	2019	1961	1285	621	819	802	546	233

Notes: Study 1 compared flood victims in the US and Brazil. Study 2 compared flood victims in the US and South Africa. Intercept is omitted from the table. Columns 1-2 and 5-6 use full sample of White respondents. Columns 3-4 and 7-8 subset to self-identified White Democrats and Republicans. Heteroskedasticity-robust standard errors in parentheses. P-values obtained using randomization inference are in square brackets. $^{***}p < 0.001$; $^{**}p < 0.01$; $^{*}p < 0.05$

C.4 Emotional attribution

Table A7. Coefficients from bivariate regressions of race and nationality treatments on selection of emotional adjectives.

	Study 1		Study 2	
	Scared	Angry	Scared	Angry
Global South	0.06**	0.00	0.00	0.00
	(0.02)	(0.01)	(0.03)	(0.01)
Black (US)	-0.02	0.00	0.02	0.01
	(0.02)	(0.01)	(0.03)	(0.02)
Black (Global South)	-0.04*	0.02	0.02	0.03*
	(0.02)	(0.01)	(0.03)	(0.02)

Notes: Study 1 compared flood victims in the US and Brazil. Study 2 compared flood victims in the US and South Africa. Each row presents coefficients from the regression of specific emotions (selected==1) on the Global South or race treatments. Intercept, R2, and number of observations are omitted from the table. Heteroskedasticity-robust standard errors in parentheses. $^{***}p < 0.001$; $^{**}p < 0.01$; $^{*}p < 0.05$

C.5 Mediation analysis: Race treatment

For the mediation analysis, we focus on White Republicans in the US condition, as the effects of race in this subsample are statistically significant at 5% in Study 2 and directionally similar, though insignificant, in Study 1.

The effect of the Black treatment is statistically significant at 10% in Study 1 when using non-robust standard errors. The mediation package uses non-robust standard errors in the underlying regressions, which partly explains why the ACME and ADE are statistically significant in some models for Study 1.

In both studies, the social proximity mechanism appears to account for much of the treatment effect, nearly the entire effect in Study 1 and half the total effect in Study 2 (see Tables A8 and A9). We also find some support for the role of perceived deservingness in reducing generosity toward racial outgroups, but only in (the larger) Study 1.

Evidence for the paternalism channel is less straightforward. The AMCE for perceived competence in Study 1 is positive and statistically significant, suggesting that the Black treatment increases generosity by evoking paternalism. However, the positive effect via the perceived competence channel is offset by the larger negative effect of the race treatment through the social proximity and deservingness channels. Thus, although White Republicans may feel some paternalism toward US Blacks, they also view this group as less deserving of aid and more socially proximate, which could explain the overall null effect on their willingness to assist Black victims. The AMCE for competence in Study 2 is null, possibly due to its smaller sample size.

Table A8. Mediation analysis for the effect of the race treatment among US branch, White Republican subsample (Study 1).

	Deservingness		Social proximity		Paternalism	
	(1)	(2)	(1)	(2)	(1)	(2)
Black Treatment	-0.05	-0.17^{***}	-0.00	-0.37^{***}	-0.15^{***}	0.11^{***}
	(0.05)	(0.06)	(0.06)	(0.07)	(0.06)	(0.04)
Mechanism	0.33^{***}		0.27^{***}		0.35^{***}	
	(0.03)		(0.03)		(0.06)	
ACME	-0.06^{***}		-0.10^{***}		0.04^{***}	
	[-0.11,-0.02]		[-0.14,-0.06]		[0.01,0.06]	
ADE	-0.05		0.00		-0.15^{**}	
	[-0.13,0.05]		[-0.11,0.11]		[-0.26,-0.01]	
R^2	0.16	0.03	0.14	0.07	0.08	0.03
Num. obs.	621	621	621	621	250	250

$^{***}p < 0.001$; $^{**}p < 0.01$; $^{*}p < 0.05$

*Notes:*Each row presents coefficients from the regression of specific emotions (selected==1) on the Global South or race treatments. Intercept, R2, and number of observations are omitted from the table. Heteroskedasticity-robust standard errors in parentheses. $^{***}p < 0.001$; $^{**}p < 0.01$; $^{*}p < 0.05$

Table A9. Mediation analysis for the effect of the race treatment among US branch, White Republican subsample (Study 2).

	Deservingness		Social proximity		Paternalism	
	(1)	(2)	(1)	(2)	(1)	(2)
Black Treatment	-0.19^{**}	-0.05	-0.10	-0.37^{***}	-0.21^{**}	0.02
	(0.09)	(0.11)	(0.09)	(0.14)	(0.09)	(0.07)
Mechanism	0.30^{***}		0.27^{***}		0.25^{***}	
	(0.05)		(0.03)		0.009	
ACME	0.01		-0.10^{**}		0.01	
	[-0.09,0.05]		[-0.19,-0.03]		[-0.03,0.03]	
ADE	-0.20^{**}		0.11		-0.20^{**}	
	[-0.39,-0.01]		[-0.22,0.08]		[-0.39,-0.04]	
R^2	0.18	0.06	0.22	0.09	0.09	0.02
Num. obs.	233	233	233	233	233	233

$^{***}p < 0.001$; $^{**}p < 0.01$; $^{*}p < 0.05$

*Notes:*Each row presents coefficients from the regression of specific emotions (selected==1) on the Global South or race treatments. Intercept, R2, and number of observations are omitted from the table. Heteroskedasticity-robust standard errors in parentheses. $^{***}p < 0.001$; $^{**}p < 0.01$; $^{*}p < 0.05$

C.6 Results by individual attitudes

Table A10. Models displaying the effect of Global South treatment on attitudes towards corruption and blameworthiness, the underlying variables for the deservingness index.

	Study 1		Study 2	
	Corrupt	Blameworthy	Corrupt	Blameworthy
Brazil	0.13^{**}	0.03		
	(0.05)	(0.05)		
South Africa			0.13	0.08
			(0.07)	(0.07)
R^2	0.04	0.02	0.07	0.04
Num. obs.	1862	1862	810	810

$^{***}p < 0.001$; $^{**}p < 0.01$; $^{*}p < 0.05$

C.7 Main results: Race treatment

Table A11. Effect of race treatment on attitudes

	Brazil (Study 1)		South Africa (Study 2)	
	(1)	(2)	(1)	(2)
**Panel A: Corrupt**				
Black Treatment	0.04	-0.05	-0.10	-0.14*
	(0.04)	(0.05)	(0.07)	(0.07)
R^2	0.02	0.05	0.08	0.06
Num. obs.	1922	1845	801	763
**Panel B: Blameworthy**				
Black Treatment	-0.07	-0.02	-0.14*	-0.09
	(0.04)	(0.05)	(0.07)	(0.07)
R^2	0.02	0.02	0.04	0.05
Num. obs.	1922	1845	801	763
**Panel C: In-Kind Aid**				
Black Treatment	0.04	0.03	-0.00	0.02
	(0.05)	(0.04)	(0.08)	(0.07)
R^2	0.00	0.00	0.02	0.01
Num. obs.	1922	1845	801	763
**Panel D: Less Capable**				
Black Treatment	0.10*	-0.02	-0.01	0.14*
	(0.04)	(0.05)	(0.07)	(0.07)
R^2	0.04	0.05	0.03	0.05
Num. obs.	1922	1845	801	763
**Panel E: Less Educated**				
Black Treatment	0.04	0.14**	-0.05	0.26***
	(0.05)	(0.04)	(0.07)	(0.07)
R^2	0.01	0.01	0.02	0.02
Num. obs.	1922	1845	801	763

Notes: Study 1 compared flood victims in the US and Brazil. Study 2 compared flood victims in the US and South Africa. Columns marked (1) refer to the effects within the US victim arm, and columns marked (2) refer to the effects within the Global South victim arm. Heteroskedasticity-robust standard errors in parentheses. P-values obtained using randomization inference are in square brackets. ***p<0.001; **p<0.01; *p<0.05.

D Robustness checks

Table A12. Effect of Global South treatment on support for governmental and personal aid in a subset of respondents who passed the attention check.

	**Panel A.** DV: Support for governmental aid					
	Study 1			Study 2		
	White	Dem.	Rep.	White	Dem.	Rep.
Brazil	-0.71***	-0.67***	-0.80***			
	(0.04)	(0.04)	(0.06)			
South Africa				-0.77***	-0.62***	-1.09***
				(0.06)	(0.06)	(0.12)
Male	-0.11***	-0.04	-0.10	-0.04	-0.01	0.05
	(0.04)	(0.04)	(0.06)	(0.05)	(0.05)	(0.11)
Income scale	-0.02***	-0.01	-0.02	-0.04***	-0.03**	-0.01
	(0.01)	(0.01)	(0.02)	(0.01)	(0.01)	(0.02)
College	-0.01	-0.03	-0.07	0.10*	0.02	0.14
	(0.04)	(0.04)	(0.07)	(0.06)	(0.06)	(0.12)
No int'l travel	0.00	0.05	-0.07	0.00	-0.05	0.29**
	(0.04)	(0.04)	(0.07)	(0.06)	(0.06)	(0.12)
R^2	0.20	0.24	0.24	0.24	0.22	0.36
Num. obs.	1723	1115	571	670	469	181
	**Panel B.** DV: Willingness to personally assist					
	Study 1			Study 2		
	White	Dem.	Rep.	White	Dem.	Rep.
Brazil	-0.23***	-0.14***	-0.37***			
	(0.04)	(0.04)	(0.06)			
South Africa				-0.28***	-0.13**	-0.59***
				(0.06)	(0.06)	(0.12)
Male	-0.17***	-0.14***	-0.18***	-0.15***	-0.16**	-0.05
	(0.04)	(0.04)	(0.06)	(0.06)	(0.06)	(0.12)
Income scale	0.00	0.03***	-0.02	-0.03**	-0.02	-0.01
	(0.01)	(0.01)	(0.02)	(0.01)	(0.01)	(0.03)
College	0.00	0.03	-0.08	0.04	0.02	0.04
	(0.04)	(0.05)	(0.07)	(0.06)	(0.07)	(0.13)
No int'l travel	0.04	0.11**	-0.05	-0.02	-0.04	0.14
	(0.04)	(0.05)	(0.07)	(0.06)	(0.06)	(0.13)
R^2	0.04	0.03	0.09	0.05	0.03	0.13
Num. obs.	1724	1115	572	670	469	181

Notes: Intercept is omitted from the table. Study 1 compared flood victims in the US and Brazil. Study 2 compared flood victims in the US and South Africa. Heteroskedasticity-robust standard errors in parentheses. ***$p<0.001$; **$p<0.01$; *$p<0.05$.

Table A13. Effect of race treatment on support for governmental aid in a subset of respondents who passed the attention check.

	**Panel A:** DV: Support for governmental aid, Global South treatment arm					
	Study 1 (Brazil)			Study 2 (S. Africa)		
	White	Dem.	Rep.	White	Dem.	Rep.
Black Treatment	0.06	0.12	-0.03	0.06	0.14	-0.12
	(0.07)	(0.07)	(0.11)	(0.08)	(0.07)	(0.15)
Male	-0.10	-0.07	-0.01	-0.16*	-0.00	-0.20
	(0.07)	(0.08)	(0.11)	(0.08)	(0.08)	(0.15)
Income (1–10)	-0.04*	-0.01	-0.05	-0.03	-0.04*	0.01
	(0.02)	(0.02)	(0.03)	(0.02)	(0.02)	(0.04)
College-Educated	0.24**	0.04	0.18	0.09	0.02	-0.10
	(0.08)	(0.09)	(0.12)	(0.09)	(0.08)	(0.16)
No int'l travel	-0.02	0.09	-0.15	-0.16	-0.13	-0.05
	(0.07)	(0.08)	(0.13)	(0.08)	(0.08)	(0.15)
R^2	0.03	0.02	0.03	0.02	0.03	0.02
Num. obs.	571	345	213	480	324	140
	**Panel B:** DV: Support for governmental aid, US treatment arm					
	Study 1 (US)			Study 2 (US)		
	White	Dem.	Rep.	White	Dem.	Rep.
Black Treatment	0.00	0.03	-0.07	-0.06	0.08	-0.03
	(0.04)	(0.05)	(0.08)	(0.10)	(0.11)	(0.17)
Male	-0.12**	-0.00	-0.10	-0.05	0.09	-0.04
	(0.04)	(0.05)	(0.08)	(0.10)	(0.11)	(0.15)
Income (1–10)	-0.02*	-0.01	-0.02	-0.00	-0.01	0.06
	(0.01)	(0.01)	(0.02)	(0.02)	(0.02)	(0.03)
College-Educated	-0.02	-0.05	-0.08	-0.09	0.03	-0.33
	(0.05)	(0.05)	(0.09)	(0.11)	(0.13)	(0.17)
No int'l travel	-0.08	-0.00	-0.10	-0.15	-0.03	-0.16
	(0.05)	(0.05)	(0.08)	(0.10)	(0.11)	(0.16)
R^2	0.01	0.00	0.01	0.01	0.01	0.06
Num. obs.	1321	872	424	285	173	110

Notes: Intercept is omitted from the table. Study 1 compared flood victims in the US and Brazil. Study 2 compared flood victims in the US and South Africa. Heteroskedasticity-robust standard errors in parentheses. $^{***}p<0.001$; $^{**}p<0.01$; $^{*}p<0.05$.

E Additional results

Table A14. Effect of Global South treatment on the victims' perceived national origin.

	Study 1	Study 1	Study 1	Study 2
	Foreign	American	Foreign	American
Brazil	0.41^{***}	-0.55^{***}		
	(0.02)	(0.02)		
South Africa			0.31^{***}	-0.57^{***}
			(0.03)	(0.03)
R^2	0.25	0.36	0.21	0.37
Num. obs.	1862	1862	753	753

Notes: Intercept and covariates omitted. Heteroskedasticity-robust standard errors in parentheses. $^{***}p < 0.001$; $^{**}p < 0.01$; $^{*}p < 0.05$

Table A15. Effect of race treatment on Baker's Perception of Foreign Poor's Agency index (introduced only in Study 2) in the South Africa treatment arm.

	DV: Perception of the foreign poor's agency		
	White	Dem.	Rep.
(Intercept)	0.19^{*}	0.26^{**}	-0.17
	(0.09)	(0.10)	(0.19)
Black Treatment	0.11	0.14^{*}	0.15
	(0.06)	(0.06)	(0.10)
Male	-0.27^{***}	-0.10	-0.42^{***}
	(0.06)	(0.06)	(0.10)
Income (1–10)	-0.02	-0.02	-0.00
	(0.01)	(0.01)	(0.03)
College-Educated	0.01	-0.01	-0.08
	(0.06)	(0.07)	(0.11)
No int'l travel	-0.06	-0.03	0.00
	(0.06)	(0.07)	(0.11)
R^2	0.04	0.02	0.08
Num. obs.	765	497	250

Notes: Heteroskedasticity-robust standard errors are shown in parentheses. $^{***}p < 0.001$; $^{**}p < 0.01$; $^{*}p < 0.05$.

Table A16. Effect of race treatment on US welfare attitudes index from Study 2 in the US treatment arm.

	DV: Support for welfare		
	White	Dem.	Rep
(Intercept)	0.28^{*}	0.42^{***}	-0.47^{*}
	(0.12)	(0.12)	(0.22)
Black Treatment	-0.07	0.01	-0.16
	(0.07)	(0.06)	(0.14)
Male	-0.15^{*}	-0.09	0.00
	(0.07)	(0.06)	(0.14)
Income (1–10)	-0.07^{***}	-0.05^{**}	-0.06^{*}
	(0.02)	(0.02)	(0.03)
College-Educated	0.20^{**}	0.08	0.08
	(0.08)	(0.07)	(0.14)
No int'l travel	-0.03	0.09	0.02
	(0.07)	(0.07)	(0.15)
R^2	0.03	0.03	0.02
Num. obs.	802	546	233

Notes: Heteroskedasticity-robust standard errors are shown in parentheses. $^{***}p < 0.001$; $^{**}p < 0.01$; $^{*}p < 0.05$.

Table A17. Effect of Global South on support for personal aid in the subset of Black respondents.

	DV: Support for governmental aid		DV: Support for personal aid	
	Study 1	Study 2	Study 1	Study 2
(Intercept)	0.60^{***}	0.52^{***}	0.15^{**}	0.18^{**}
	(0.05)	(0.06)	(0.06)	(0.07)
Brazil	-0.67^{***}		-0.20^{**}	
	(0.08)		(0.09)	
South Africa		-0.57^{***}		0.05
		(0.09)		(0.10)
R^2	0.20	0.15	0.02	0.00
Num. obs.	272	214	271	214

$^{***}p < 0.01$; $^{**}p < 0.05$; $^{*}p < 0.1$

Table A18. Effect of race treatment on support for government aid in the subset of Black respondents.

	Study 1		Study 2	
	Global South	US	Global South	US
(Intercept)	-0.07	0.60^{***}	-0.06	0.52^{***}
	(0.06)	(0.05)	(0.07)	(0.06)
Black Treatment	0.03	-0.04	0.07	0.08
	(0.09)	(0.07)	(0.11)	(0.09)
R^2	0.00	0.00	0.00	0.00
Num. obs.	282	263	217	199

$^{***}p < 0.001$; $^{**}p < 0.01$; $^{*}p < 0.05$

References

[1] Kosuke Imai, Luke Keele, Dustin Tingley, and Teppei Yamamoto. Unpacking the black box of causality: Learning about causal mechanisms from experimental and observational studies. *American Political Science Review*, 105(4):765–789, 2011. ISSN 1537-5943, 0003-0554. doi: 10.1017/S0003055411000414. https://doi.org/10.1017/S0003055411000414.
